# Diesel Fuel from Used Frying Oil

**DOI:** 10.1155/2014/683272

**Published:** 2014-01-16

**Authors:** Bronislaw Buczek

**Affiliations:** Faculty of Energy and Fuels, AGH University of Science and Technology, Mickiewicz Avenue 30, 30-059 Cracow, Poland

## Abstract

New conversion technologies of used edible oils and waste animal fats into a biofuel appropriate for use in standard diesel engines have been developed, taking into consideration environmental requirements and improvement in the economics of current trans-esterification technologies. The variation in the properties of substrates made from used rape oil after treatment with mixed adsorbents (active carbon, magnesium silicate) was studied in this work. The obtained results are compared with the quality requirements for the substrates used in Vogel & Noot GmbH technology for transesterification of oils and fats.

## 1. Introduction

Biodiesel is a trade name of the fuel obtained in the process of transesterification of vegetable oils. This method is used for the production of diesel engine fuel having the properties close to diesel oil from petroleum. The viscosity of this oil is lower than that of vegetable oil. Often the name biodiesel has been used for fatty acid methyl esters (FAME) from rape oil, which are main components of biodiesel in Europe.

The economic effectiveness of the production of fatty acid methyl esters applied in the production of the biofuel has been diminishing. One way of reducing the production costs for biodiesel fuels is the use of nonedible oils, which tend to be considerably cheaper than edible vegetable oils. The technology of simultaneous transesterification of used and fresh frying oils was industrially introduced at the end of the ninety nineties.

Recycled or waste oils have evolved as popular sources for the production of biodiesel, as they are inexpensive and offer the additional environmental benefit of using substances, which would otherwise have to be disposed of.

**Figure 1:**
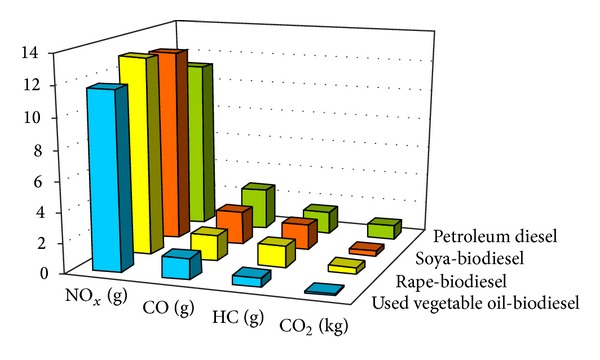
Emission of exhaust gases by trucks per one km for various fuels.

Long recognized for its environmental benefits, biodiesel is renewable, nontoxic, biodegradable, and sulphur-free, emitting 80% fewer hydrocarbons, 60% less carbon dioxide, and 50% less particulate matter than petroleum diesel. Biodiesel is 11% oxygen by weight and, therefore, burns more completely than petrodiesel. In fact, a 20% blend with petrodiesel in trucks and buses would eliminate the black smoke (actually unburned fuel) emitted during acceleration. As an example (see Figure 1), biodiesel in the form of esters from waste cooking oils was tested and it was reported that emissions were favorable.

The first article which reported on successful engine tests for methyl-, ethyl-, and 1-butylesters produced from used frying oil appeared in 1983 [1]. At the same time, recycled-frying oils were studied as raw material by Mittelbach and Remschmidt [2], who later developed a commercial process for converting waste oils from households and restaurants as well as fatty waste from slaughter houses and sewage plants into biodiesel. Since dried, mechanically purified waste oil is sold at about the half price of vegetable oils, then use of such materials makes sense from an economic point of view.

In Austria, recycled-frying oil is now an established alternative source of fatty material for the production of biofuels. Recycled-frying oil methyl esters (RFO-ME) have been commercially produced since 1992 and have been used to fuel buses serving the city of Graz. In 2004, fifty buses running on RFO-ME are covering a total annual distance of about 3,000,000 km, and in 2005, more than a hundred buses were operating on recycled-frying oil methyl esters. So far hardly any fuel related problems have been reported. The physical properties of RFO-ME slightly differ from FAME. So values for viscosity and carbon residue of recycled-frying oil methyl esters tend to be slightly higher. Moreover, the freezing point is higher than that of FAME, so in Graz, during winter, RFO-ME was blended with petroleum diesel. On the whole, the Graz city bus project is considered a sweeping success, which has found broad acceptance by the public and earned its initiators [2].

Almost all currently used technologies of vegetable oil conversion into diesel engine fuel require raw materials of high quality, among others of high triglyceride content. The classical method for obtaining methyl esters of fatty acids is based on alkaline-catalyzed transesterification. It suffers from many disadvantages. First of all this process precedes too slowly and stops before the end. Moreover, it cannot be used in the case of substrates having high free fatty acid content, which neutralize the alkaline solution and form soaps.

Recently new technologies have been introduced, which make the conversion of used edible oils and waste animal fats to the fuel appropriate for diesel engines possible, following all requirements of environmental protection and economical improvement. In all the cases these technologies require the substrates derived from oils or waste fats of defined physical and chemical properties [3–5].

This work presents the results of the studies on the changes in the properties of the substrate derived from used oil after treatment with a two-component mixture of various adsorbents.

## 2. Experimental Procedure

### 2.1. Materials

Active carbon (AR) (0.063–0.125 mm) was originally obtained from charcoal, activated using steam, and produced by ELBAR-Katowice Sp. z o. o. Carbon Raciborz, Poland.

Magnesium silicate (MG) Florisil (60–100 mesh) was produced by Fluka AG, Buchs SG, Switzerland.

**Figure 2:**
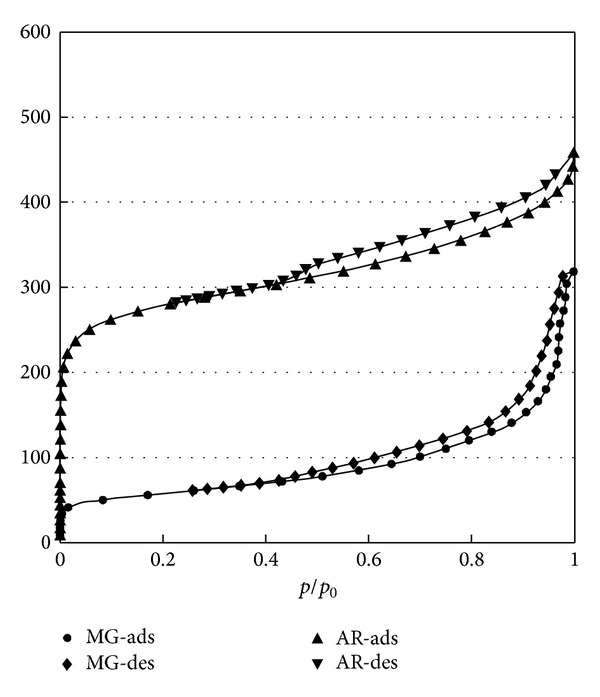
Nitrogen adsorption isotherms on MG and AR adsorbents.

Their porous structure was analysed on the basis of low-temperature (77 K) nitrogen adsorption-desorption isotherms (Figure 2).

Fresh universal oil (UO), refined rapeseed oil with low erucic acid content, was obtained from WZT ADM Szamotuły Sp. z o. o., Poland.

Used universal oil (UU) after frying French chips in a deep fryer at the temperature of about 175°C for 24 hours was obtained from one of the restaurants in Cracow.

From the obtained isotherms, such parameters were determined which characterised the micro- and mesoporous structure (*S*_mi_, *W*_*o*_, *S*_me_, *V*_me_) and the surface area (*S*_BETs_). These parameters are listed in Table 1.

### 2.2. Purification of Used Frying Oil

The oil sample was obtained by treatment with dried active carbon (50%) and magnesium silicate (50%) in a weight ratio of 15 : 1. Oil UU and a mixture of adsorbents were stirred and heated for 30 minutes at the temperature of 70–80°C. After completion of this process adsorbents were separated from the oil through filtration of the hot suspension (about 60°C) in a filtration apparatus. Filtrated purified oil, so-called UT, was thus obtained.

## 3. Results

The oil purification process aimed to remove degradation products, which were formed during frying of food, and to evaluate efficiency of applied mixture of adsorbents. To assess the changes in oil properties upon frying and purifying, the values of the following parameters were determined:

 density, *ρ* ^20^ (PN-EN ISO 12185:2002),
 viscosity, *ν* ^40^ (PN-EN ISO 3104:2004),
 flow point, *t*_*f*_ (PN-83/C-04117),
 water content, *w* (PN-EN ISO 12937:2005),
 acid value, AV (PN-85/C-04066),
 iodine value, IV (PN-ISO 3961:1998),
 sulphur content, SC (PN ISO 20884:2004).

The polymer content, PC, in oil samples was determined using Food Oil Monitor 200, an oil quality meter [6]. The results are summarized in Tables 2 and 3.

## 4. Conclusions

Communication presents the results of investigations on the possibility of use of waste oils, mainly rapeseed, as raw material for the production of fatty acid methyl esters. A number of physical and chemical properties of fats obtained gastronomy, and different oils and fats remaining after frying frozen food [7, 8].

Table 4 shows the quality requirements for the raw materials used in Vogel & Noot Patent of oil and fat transesterification.

The mixture of adsorbents applied for purification, that is, active carbon and magnesium silicate, appeared to be promising in regard to changing the properties of the raw material used for transesterification reaction.

Adsorptive treatment of the raw material before trans esterification reaction requires the use of a mixture of adsorbents having different physic-chemical properties [10, 11].

Water, FFA, polymers, and sulphur contents are decreased upon adsorption treatment, whereas iodine values do not change.

After adsorption of the impurities from used oil, the flow point, which is close to the freezing point, increases significantly.

A number of problems still remain to be analyzed regarding this technology and I hope to discuss some of them in the extended version of this paper.

## Figures and Tables

**Table 1:** Physicochemical properties of adsorbents used.

Parameter	AR	MG
BET surface area, *S*_BET_, m^2^ g^−1^	980	198
Volume of micropores, *W*_*o*_, cm^3^ g^−1^	0.42	—
Volume of mesopores, *V*_me_, cm^3^ g^−1^	0.20	0.40
Mesopore surface area, *S*_me_, m^2^ g^−1^	207	159
Micropore surface area, *S*_mi_, m^2^ g^−1^	660	—

**Table 2:** Physicochemical properties of fresh, used, and treated oils.

Oils	*ρ* ^20^ (g/cm^3^)	*ν* ^40^ (mm^2^/s)	*t*_*f*_ (°C)	W (%)
UO	0.9165	35.40	−19	<0.03
UU	0.9199	41.89	−12	<0.03
UT	0.9222	42.07	+19	204 ppm

**Table 3:** Quality indicators of fresh, used, and treated oils.

Oils	AV (mgKOH/g)	IV (gI_2_/100 g)	SC (mg/kg)	PC (%)
UO	0.81	115.1	6.9	1.5
UU	0.66	98.3	8.4	6
UT	0.44	96.7	7.6	3

**Table 4:** Requirements for Vogel & Noot GmbH process.

Requirements	Alkaline catalyst
Water content, wt.%	0.5
FFA, wt.%	3
Polymers, wt.%	2
Iodine value, IV	105
Freezing point, °C	50
Sulphur content, wt.%	0.02

Source: [9].
